# LGALS3 marks a pro-inflammatory, efferocytosis-low hepatic macrophage state and promotes hepatic stellate cell activation through the candidate receptor ANXA2 in liver cirrhosis

**DOI:** 10.3389/fimmu.2026.1891906

**Published:** 2026-09-21

**Authors:** Fengchun Li, Hongliang Chen, Jihan Qi, Jiaxin Wang, Lingyi Xu, Zhuo Yu, Ram Prasad Chaulagain, Cuihua Li, Xin Ni, Shizhu Jin

**Affiliations:** 1 Department of Gastroenterology and Hepatology, The Second Affiliated Hospital, Harbin Medical University, Harbin, Heilongjiang, China; 2 Digestive Endoscopic Center, The Second Affiliated Hospital, Harbin Medical University, Harbin, Heilongjiang, China

**Keywords:** annexin A2, efferocytosis, galectin-3, inflammatory macrophages, liver cirrhosis

## Abstract

**Introduction:**

Efferocytosis is a macrophage-dependent process responsible for clearing apoptotic cells. Reduced efferocytic capacity may be one of the factors contributing to liver cirrhosis (LC). This research aimed to identify and validate efferocytosis-related genes (EFRGs) associated with LC and investigate their potential roles in LC pathogenesis.

**Methods:**

Bulk RNA sequencing (bulk RNA-seq) analysis was performed to identify LC-assoated differentially expressed EFRGs and assess alterations in the immune microenvironment. Machine learning models combined with Shapley Additive Explanations (SHAP) analysis were used to screen hub genes. Single-cell RNA sequencing (scRNA-seq) analysis was performed to determine the cellular localization of galectin-3 (LGALS3) and explore the differences in efferocytic capacity among macrophage subpopulations and the potential involvement of LGALS3. NicheNet analysis and molecular structure modelling were used to screen candidate receptors, with further prioritization based on gene expression patterns. Immunohistochemistry (IHC), immunofluorescence (IF), and western blot (WB) were further used to validate hub gene expression and efferocytic activity and explore the downstream mechanism of the hub gene. To further evaluate the role of macrophage-derived LGALS3 in hepatic stellate cell (HSC) activation, LGALS3 was silenced by siRNA in polarised inflammatory macrophages before Transwell co-culture with JS1 cells.

**Results:**

LGALS3 was identified as a hub EFRG positively associated with LC. LGALS3 expression tended to increase during liver fibrosis progression and was significantly higher in human and mouse cirrhotic tissues than in normal liver tissues. Moreover, LGALS3 expression was associated with immune infiltration characteristics in LC samples. scRNA-seq analysis revealed that LGALS3 was localized mainly to inflammatory macrophages and was significantly upregulated in this macrophage subset in LC tissues. LGALS3 marks a pro-inflammatory, low efferocytic capacity hepatic macrophage state. Based on gene expression pattern, together with molecular dynamics simulations, and experimental validation prioritised annexin A2 (ANXA2) as a candidate receptor potentially involved in LGALS3-mediated HSC activation. Consistently, silencing LGALS3 in inflammatory macrophages attenuated the upregulation of ANXA2 and α-SMA in co-cultured JS1 cells.

**Conclusions:**

As a key EFRG in LC pathogenesis, LGALS3 marked a pro-inflammatory hepatic macrophage state with low efferocytic capacity and promoted HSC activation, potentially through ANXA2 as a candidate receptor for inflammatory macrophage-derived LGALS3.

## 1 Introduction

Liver cirrhosis (LC) is a significant global health concern (1, 2). Liver transplantation, as a curative treatment for patients with advanced LC, is limited by a shortage of donor organs (3, 4). Therefore, exploring the pathogenic mechanisms of LC to identify targets for early diagnosis and treatment is critical.

LC is not simply a fibrotic change in liver structure; the underlying causes of acute decompensation and multiorgan failure are chronic systemic inflammation and the resulting complex immune-inflammatory dysfunction (5, 6). Moreover, immune cell dysfunction is closely associated with disease severity, with impaired neutrophil and macrophage function markedly increasing susceptibility to infection and organ failure (7). Among these, macrophage dysfunction is particularly critical, as macrophages not only regulate the inflammatory response and fibrotic remodelling in chronic liver injury but also mediate the removal of apoptotic cells (8).

Efferocytosis, the clearance of apoptotic cells, is essential for tissue homeostasis. During the progression of LC, impaired efferocytic capacity may lead to the persistent accumulation of dead cells, thereby exacerbating inflammatory responses, compromising reparative programs, and promoting fibrogenesis (9). Therefore, elucidating the molecular mechanisms underlying defective efferocytic capacity in the context of LC is highly important. Hepatic macrophages are the principal effector cells of efferocytosis in the liver (9). They drive chronic liver injury and represent potential targets for antifibrotic therapy (10). Different macrophage subsets play distinct or even opposing roles in promoting the progression of cirrhosis (10). Therefore, understanding the functions of macrophage subpopulations may provide new insights into the development of future treatment strategies for cirrhosis.

Our systematic analysis based on bulk RNA sequencing (bulk RNA-seq) data combined with experimental validation using human and mouse liver tissuesidentified galectin-3 (LGALS3) as a hub efferocytosis-related gene (EFRG). Single-cell RNA sequencing (scRNA-seq) was subsequently performed to evaluate the expression profile of LGALS3 in macrophage subpopulations and to investigate its potential role in phenotypic changes in macrophages with high and low efferocytic capacity. To further elucidate the potential mechanisms by which LGALS3 promotes hepatic stellate cell (HSC) activation and drives the progression of LC, this study conducted a systematic investigation integrating ligand-receptor interaction predictions, molecular structure modelling, and multilevel experimental validation. The experimental flowchart is shown in **Figure 1**.

**Figure 1:**
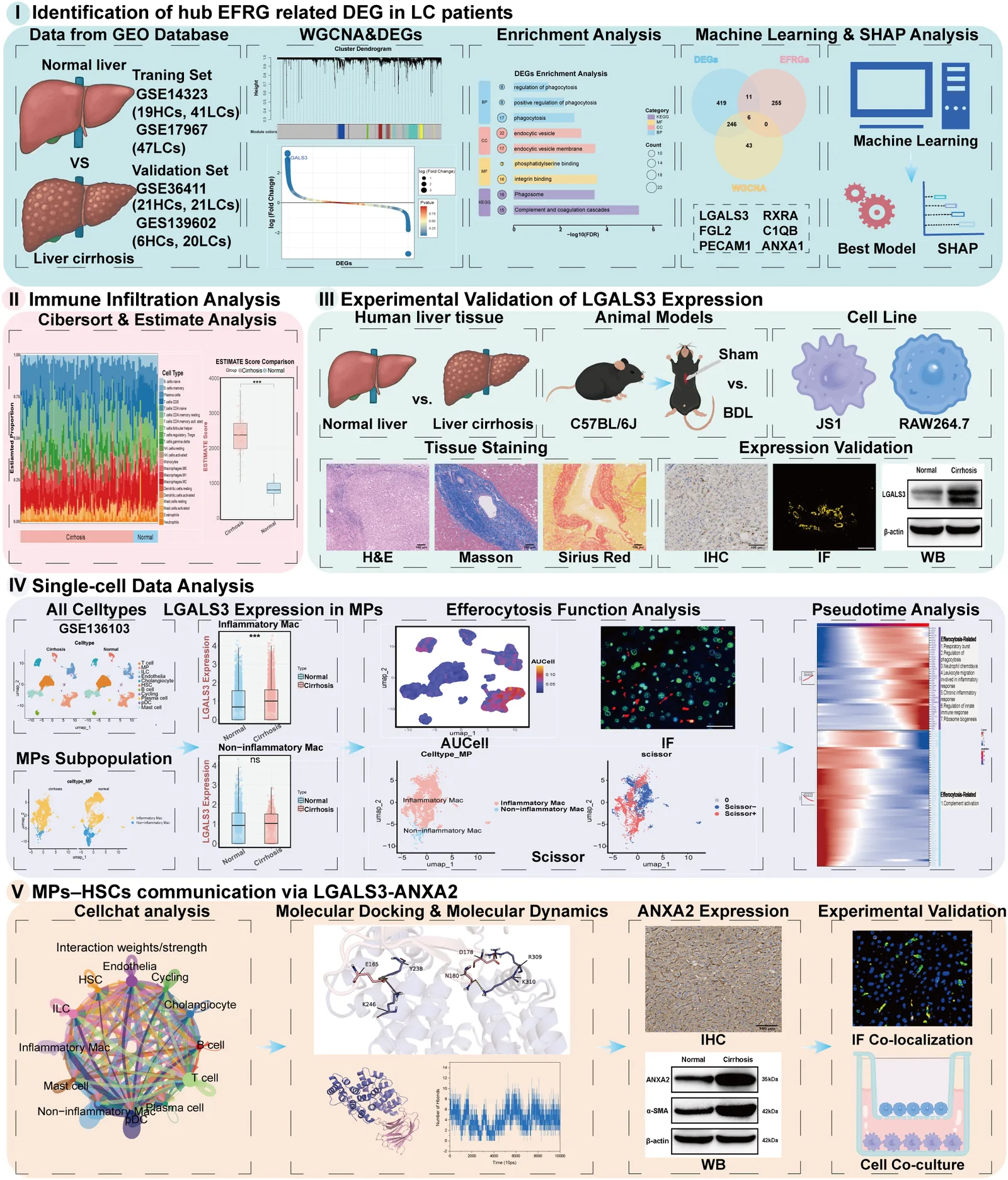
Flowchart of the study.

## 2 Materials and methods

### 2.1 Data collection and preprocessing

#### 2.1.1 Bulk RNA-seq datasets

Five bulk RNA-seq datasets were used in this study (**Supplementary Table S1**), including four microarray datasets (GSE14323, GSE17967, GSE36411, and GSE139602) and one high-throughput sequencing dataset (GSE276114), all of which were obtained from the GEO database. The expression sets GSE14323 and GSE17967 were combined (19 normal samples and 88 cirrhotic samples) and utilized as the training set, and the GSE36411 and GSE139602 datasets were combined (27 normal samples and 41 cirrhotic samples) and utilized as the validation set. The GSE276114 dataset was further employed to investigate hub gene expression dynamics during liver fibrosis progression.

#### 2.1.2 scRNA-seq datasets

The scRNA-seq dataset GSE136103 (5 normal samples and 5 cirrhotic samples) (**Supplementary Table S1**), was processed using the “Seurat” R package (version 5.3.0). Cells with 300-6, 000 detected genes, a mitochondrial gene content less than 20%, and a haemoglobin gene content less than 5% were retained for downstream analysis. After Harmony-based batch correction, principal component analysis, clustering, and uniform manifold approximation and projection (UMAP) visualization were performed.

#### 2.1.3 Gene set retrieval

A total of 272 EFRGs (**Supplementary Table S2**) were obtained from the study by Chenghui Cao et al. (11). A total of 463 extracellular matrix (ECM)-related genes (**Supplementary Table S3**) were obtained from the study by Xinhao Shao et al. (12).

### 2.2 Bulk RNA-seq data analysis

#### 2.2.1 Weighted gene co-expression network analysis

WGCNA was performed on the merged training set using the R package “WGCNA” (version 1.73). Genes were first filtered by variance, and the top 25% were retained. The soft-thresholding power for scale-free network construction was determined using the “pickSoftThreshold” function (R² ≥ 0.9), followed by module detection with the “blockwiseModules” function. Module eigengenes (MEs), representing the first principal component of each module, were ordered using the “orderMEs” function. Finally, Pearson correlations between MEs and clinical phenotypes were computed to identify modules significantly associated with LC.

#### 2.2.2 Identification and functional enrichment analysis of differentially expressed genes

The “limma” R package (version 3.64.1) was used to identify DEGs. DEGs with logFC > 0.8 and *P* value < 0.05 were defined as upregulated DEGs, whereas those with logFC < −0.8 and *P* value < 0.05 were defined as downregulated DEGs.

The “clusterProfiler” R package (version 4.16.0) was used to perform functional enrichment analysis on both types of DEGs mentioned above. Gene Ontology (GO) and Kyoto Encyclopedia of Genes and Genomes (KEGG) enrichment analyses were performed.

#### 2.2.3 Identification of candidate efferocytosis-related DEGs

Candidate genes were screened by integrating the DEGs, EFRGs, and module genes identified by WGCNA. The “ggvenn” R package (version 0.1.19) was used to create a Venn diagram showing intersecting genes. The “RCircos” R package (version 1.2.2) was used to show the locations of the candidate genes on human chromosomes. The “corrplot” R package (version 0.95) was used to calculate correlations among the candidate genes.

#### 2.2.4 Machine learning

To select the optimal model for identifying candidate genes for LC diagnosis, we trained 8 machine learning models, namely, random forest, linear discriminant analysis (LDA), gradient boosting machine, partial least squares, k-nearest neighbours, extreme gradient boosting tree, least absolute shrinkage and selection operator and light gradient boosting machine (LightGBM), on the training set. For each algorithm, we employed 5-fold cross-validation for model training and hyperparameter tuning using the R packages “caret” (version 7.0-1) and “lightgbm” (version 4.6.0). Model performance was verified using the validation set after training was complete.

The R package “pROC” (version 1.19.0.1) was used to generate receiver operating characteristic (ROC) curves, calculate the area under the ROC curve (AUC) and the 95% confidence interval (CI), and plot the ROC curves and forest plots.

The final LDA model was internally validated using 1, 000 stratified bootstrap resamples. Mean optimism was calculated by comparing the AUC in each bootstrap sample with that in the original training set and was subtracted from the apparent AUC to obtain the optimism-corrected AUC. The six predictors remained fixed throughout the bootstrap analysis.

#### 2.2.5 SHapley Additive exPlanations

SHAP was employed to analyse the best-performing model, with the aim of identifying the gene with the greatest influence on LC prediction (13). SHAP values were computed using the “kernelshap” R package (version 0.9.1) if the best-performing model was trained with caret or using the “fastshap” R package (version 0.1.1) if trained with LightGBM.

#### 2.2.6 Immune infiltration analysis

Two types of immune infiltration analyses were employed, namely, CIBERSORT and ESTIMATE. The “CIBERSORT” R package (version 0.1.0) was used to estimate the differences in the abundance proportions of immune cells between the normal and cirrhotic samples. The “ESTIMATE” R package (version 1.0.13) was used to calculate the differences in the ESTIMATE, immune and stromal scores between the normal and cirrhotic samples.

### 2.3 scRNA-seq data analysis

#### 2.3.1 Cell type annotation

scRNA-seq data were analysed utilizing the “Seurat” R package (version 5.3.0). The cell markers employed (**Supplementary Table S4**) were obtained from the studies by P Ramachandran et al. and Sonya A. MacParland et al. (14, 15). Cells were annotated according to the average expression levels of specific markers across all clusters.

#### 2.3.2 Validation of hub gene expression in the scRNA-seq data

The “FindAllMarkers” function was used to analyse genes with differential expression between cell populations. The “VlnPlot” function was used to visualize cell type-specific differences in LGALS3 expression between the normal and cirrhotic groups.

#### 2.3.3 CellChat analysis

CellChat analyses were performed to explore cell-cell communication and related ligand-receptor interactions. The “CellChat” R package (version 1.6.1) was used to evaluate the intercellular communication networks. The “nichenetr” R package (version 2.2.1) was used to identify candidate receptors on HSCs that interact with the ligand LGALS3 released by mononuclear phagocytes (MPs) and subsequently influence the transition of HSCs from a normal state to a cirrhotic state.

#### 2.3.4 Pseudotime analysis

Pseudotime analysis was employed to map the differentiation trajectories of various macrophage types and track the corresponding changes in gene expression during these processes. The “monocle” R package (version 2.36.0) was used to perform pseudotime analysis. The top 100 genes associated with pseudotime were selected for enrichment analysis and visualization using “jjAnno” (version 0.0.3) and “org.Hs.eg.db” (version 3.21.0).

#### 2.3.5 AUCell analysis

The “AUCell” R package (version 1.30.1) was used to quantify the efferocytic capacity and ECM deposition activity of the MPs under different grouping conditions. The gene sets used were based on EFRGs and ECM-related genes. Newly classified subgroups were defined on the basis of the median level. The UMAP algorithm was used to visualize efferocytic activity across different cell populations.

#### 2.3.6 Scissor analysis

The “GSVA” R package (version 2.2.0) was applied to score the cirrhotic samples in the training set on the basis of gene sets composed of EFRGs. Scores > 0 were classified as high efferocytic capacity, and scores ≤ 0 were classified as low efferocytic capacity. The “Scissor” R package (version 2.0.0) was subsequently used to integrate the scRNA-seq data with the bulk RNA-seq data to identify cell subclusters associated with the phenotypes (high/low efferocytic capacity) in LC tissue. To assess potential circularity, the AUCell and Scissor analyses were repeated using the efferocytosis-related gene set after excluding LGALS3, and the results were compared with those obtained using the original gene set.

### 2.4 AlphaFold complex structure prediction and molecular dynamics simulations

#### 2.4.1 AlphaFold complex structure prediction

Protein sequences were retrieved from the UniProt database (https://www.uniprot.org/). The complex structures of LGALS3 with the candidate receptors ANXA2, PTPRK, ENG, and MCAM were predicted using the AlphaFold Server (https://alphafoldserver.com/welcome), and the corresponding pTM and ipTM scores were obtained from the server outputs. The predicted protein–protein complex structures were imported into Maestro Elements 6.1 for structural optimization and subsequently visualized using PyMOL. PRODIGY

(https://wenmr.science.uu.nl/prodigy/) was used to estimate the binding energy from the predicted complex structures.

#### 2.4.2 Molecular dynamics simulations between LGALS3 and Annexin A2

The AlphaFold-derived protein-protein complex was subjected to a 100-ns simulation in AMBER 22 with the ff14SB force field. The system was placed in a truncated octahedral TIP3P water box with 10 Å of buffer and neutralized using Na^+^/Cl^-^ ions. After minimization, heating, and NVT/NPT equilibration, a production simulation was performed under periodic boundary conditions. PME, SHAKE, and the Langevin algorithm were used for electrostatics, hydrogen-bond constraints, and temperature control, respectively. Trajectories were analysed using PyMOL and Origin 2021.

### 2.5 Experimental validation

#### 2.5.1 Clinical sample collection

Liver tissue samples were obtained from the Second Affiliated Hospital of Harbin Medical University with ethical approval and informed consent (Approval No. KY2025-095), comprising normal liver tissues adjacent to hepatic haemangiomas (n = 3) as healthy controls and cirrhotic liver tissues adjacent to hepatocellular carcinoma (HCC) (n = 3) as the disease/LC group (**Supplementary Table S5**).

#### 2.5.2 Establishment of a mouse bile duct ligation model

Seven-week-old male C57BL/6J mice were provided by the Animal Center of the Second Affiliated Hospital of Harbin Medical University. After one week of acclimatization (at 8 weeks of age), BDL was established by ligating the common bile duct under tribromoethanol anaesthesia, while sham mice underwent laparotomy without ligation. Four weeks after surgery (at 12 weeks of age), all mice were euthanized, and liver tissues were collected for subsequent analyses (**Supplementary Figure S1**). Three mice were included in each group (n = 3 per group), with no post-operative mortality in either group. All experimental procedures involving animals were approved by the institutional ethics committee (Approval No. SYDW2026-015).

#### 2.5.3 RAW264.7 cells polarization

RAW264.7 (Servicebio, Wuhan, China) cells were cultured in fresh complete medium at 37 °C with 5% CO_2_. RAW264.7 cells were stimulated with either IFN-γ (20 ng/mL) plus LPS (100 ng/mL) or IL-4 (20 ng/mL) for 24 h to induce inflammatory or non-inflammatory macrophage phenotypes (16, 17), respectively. Successful macrophage polarization was confirmed by western blot (WB) of the inflammatory macrophage marker CD86 and the non-inflammatory macrophage marker ARG1.

#### 2.5.4 siRNA transfection

To identify an effective sequence for gene silencing, inflammatory macrophages were separately transfected with three small interfering RNAs targeting LGALS3 or a negative control siRNA (**Supplementary Table S6**) (18) using Lipofectamine RNAiMAX reagent (Invitrogen, Carlsbad, CA, USA). The final siRNA concentration was 50 nM. The knockdown efficiency was evaluated by WB analysis, and the siRNA that resulted in the greatest reduction in LGALS3 expression was selected for subsequent experiments.

#### 2.5.5 Cell co-culture

JS1 cells (Otwo Biotech, Guangzhou, China) were cultured in fresh complete medium at 37 °C with 5% CO_2_. For co-culture experiments, inflammatory or non-inflammatory macrophages were seeded in the upper chambers, while JS1 cells were seeded in the lower chambers. Fresh complete medium was added to both chambers to maintain an equal volume of liquid, and co-cultures were maintained under standard conditions for 48 h. After co-culture, the JS1 cells from the lower chambers were collected, and total and membrane proteins were extracted for subsequent WB analysis.

#### 2.5.6 Histological staining

Human and mouse liver specimens were fixed with 4% paraformaldehyde, embedded in paraffin, and cut into 3-μm sections. Consecutive sections were stained with H&E, Masson, or Sirius Red, sealed with neutral resin, and examined using light microscopy.

#### 2.5.7 Immunohistochemistry

Liver paraffin sections were deparaffinized, rehydrated, blocked for endogenous peroxidase activity, subjected to antigen retrieval and BSA blocking, and then incubated with primary antibodies overnight at 4 °C with anti-LGALS3 antibody (1:50, 250013, Zen Bioscience Co., Ltd.) or anti-ANXA2 antibody (1:100, R23478, Zen Bioscience Co., Ltd.), followed by incubation with goat anti-rabbit IgG (1:10, 000) for 1 h at 37 °C. DAB was used for signal detection, and the sections were counterstained with haematoxylin and observed under a light microscope.

#### 2.5.8 WB analysis

After being washed with cold PBS, the cells were lysed in RIPA buffer on ice. The lysates were sonicated, centrifuged, and quantified using a BCA assay. Equal amounts of protein were separated by 10-12% SDS-PAGE, transferred to PVDF membranes, blocked with 5% non-fat milk, and probed with primary antibodies overnight at 4 °C. The primary antibodies used were anti-LGALS3 (1:50), anti-ANXA2 (1:100), anti-α-SMA (1:100, AB5694, Abcam), anti-ARG1 (1:5000, 66129-1-Ig, Proteintech), anti-CD86 (1:1000, 13395-1-AP, Proteintech), and anti-β-actin (1:10000, 66009-1-Ig, Proteintech). After the membranes were washed, they were incubated with secondary antibodies, and protein signals were detected by enhanced chemiluminescence. Liver tissues (30 mg) were lysed in 200 μL of RIPA buffer and processed as described above.

#### 2.5.9 Immunofluorescence staining

Frozen liver sections were washed with PBS, blocked with goat serum at room temperature, and incubated overnight at 4 °C with primary antibodies against LGALS3 (1:50), ANXA2 (1:100), and F4/80 (1:200, HA721520, HUABIO). The sections were incubated with fluorescently labelled secondary antibodies for 1 h at 37 °C in the dark, followed by TUNEL staining using an *In Situ* Cell Death Detection Kit, TMR Red (Roche), according to the manufacturer’s protocol. After being washed with PBS, the sections were mounted with antifade medium and observed under a fluorescence microscope.

#### 2.5.10 Multiplex immunofluorescence-based spatial distance analysis

mIF images were analysed to assess the spatial proximity between LGALS3^+^ F4/80^+^ macrophages and ANXA2^+^ cells. DAPI-stained nuclei were used for the calculations. After thresholding, binary masks were processed using the “Fill Holes” function to restore incomplete nuclear regions, and adjacent cells were separated using the “Watershed” algorithm. The “Analyse Particles” function was subsequently used to determine the centroid coordinates (X, Y) of individual cells (19, 20). LGALS3^+^ F4/80^+^ macrophages and ANXA2^+^ cells were manually identified according to fluorescence signal localization and intensity. In each field of view, the distance between LGALS3^+^ F4/80^+^ macrophages and ANXA2^+^ cells was calculated using the “dist” function in R, and the average nearest-neighbour distance was used for statistical analysis.

### 2.6 Statistical analysis

As for bioinformatic, R (version 4.5.0) was used mainly for bulk RNA-seq and scRNA-seq data processing, statistical analysis, and visualization. Fiji (ImageJ, version 1.54p) and Experimental data were statistically analysed and visualized using GraphPad Prism (version 10.6.1). The Wilcoxon rank-sum test and t test were employed to assess whether the difference between two sets of data was statistically significant, with the specific statistical test used for each comparison indicated in the corresponding Results description. Spearman and Pearson correlation analyses were performed.

As for experimental results, n = 3 indicates that three independent mice or three independent human liver tissue specimens were used per group. Independent samples t-test for between-group comparisons were used. Technical replicates were averaged to obtain a single value for each biological sample and were not treated as independent observations in the statistical analyses. An independent-samples t-test was used for comparisons between two groups. One-way ANOVA was used for comparisons among multiple groups, including the four-group comparison of si-NC and the three si-LGALS3 constructs. All t-tests were two-tailed. *P* < 0.05 was considered to indicate statistical significance.

## 3 Results

### 3.1 Identification of candidate genes exhibiting the highest positive correlations with LC through WGCNA

We employed the WGCNA method to construct a coexpression network. During the WGCNA analysis, all genes passed the quality checks, and no outlier samples were detected; thus, no samples were removed. At a soft-thresholding power of 16 (scale-free R² = 0.9), six gene modules were identified (**Figures 2A, B**). On the basis of the module-trait relationship heatmap (**Figure 2C**), we ultimately selected the turquoise module (Pearson’s r = 0.8, *P* = 2 × 10^-^²^5^), which showed the greatest and most significant correlation with LC.

**Figure 2:**
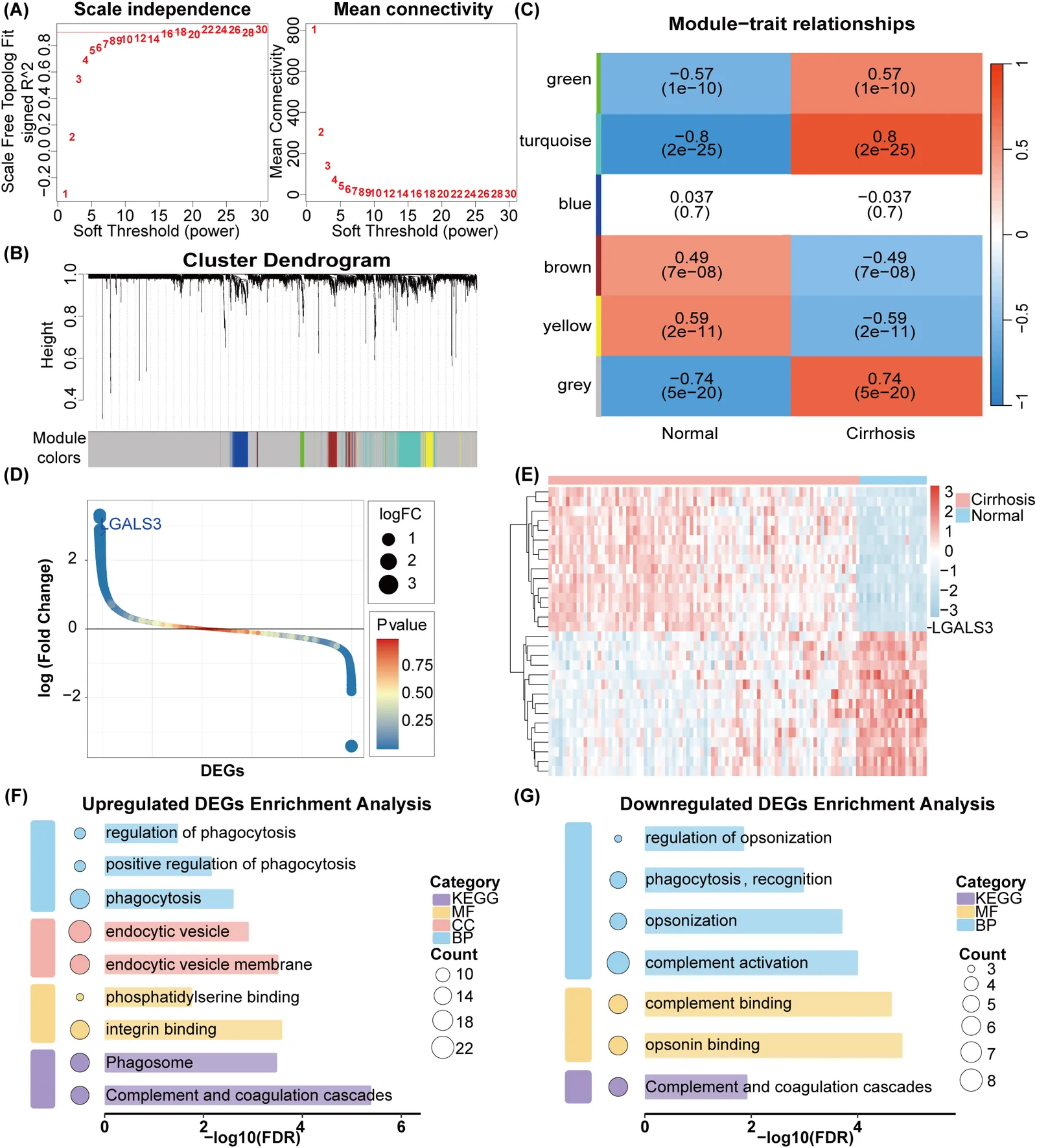
Integrated WGCNA, differential expression, and enrichment analyses identified candidate liver cirrhosis-associated DEGs and potential pathways. **(A)** Scale independence and mean connectivity for choosing the optimal soft-threshold power. **(B)** Cluster dendrogram of all genes. **(C)** Heatmap of the correlations between gene modules and traits. **(D)** Volcano plot of DEGs in the normal and cirrhotic groups. **(E)** Heatmap of DEGs in the normal and cirrhotic groups. **(F)** GO and KEGG enrichment analyses of upregulated DEGs. **(G)** GO and KEGG enrichment analyses of downregulated DEGs.

### 3.2 Identification and functional enrichment analysis of DEGs

A total of 682 DEGs were identified, including 505 upregulated DEGs and 177 downregulated DEGs (**Figure 2D**). The top 15 upregulated and downregulated DEGs were visualized using a heatmap (**Figure 2E**). Both the GO and KEGG enrichment analyses revealed significant enrichment in efferocytosis-related pathways (**Figures 2F, G**; **Supplementary Tables S7**–**S10**).

### 3.3 Identification and DNA location of efferocytosis-related DEGs

A Venn diagram was generated, which revealed that a total of 6 differentially expressed EFRGs were positively correlated with LC. These genes, namely, C1QB, FGL2, ANXA1, RXRA, LGALS3 and PECAM1, were defined as candidate genes (**Figure 3A**). These genes were mapped to chromosomes 1, 7, 9, 14, and 17 (**Supplementary Figure S2A**; **Supplementary Table S11**). Correlation analysis revealed synergistic relationships among all candidate genes, except RXRA, in LC (**Supplementary Figure S2B**).

**Figure 3:**
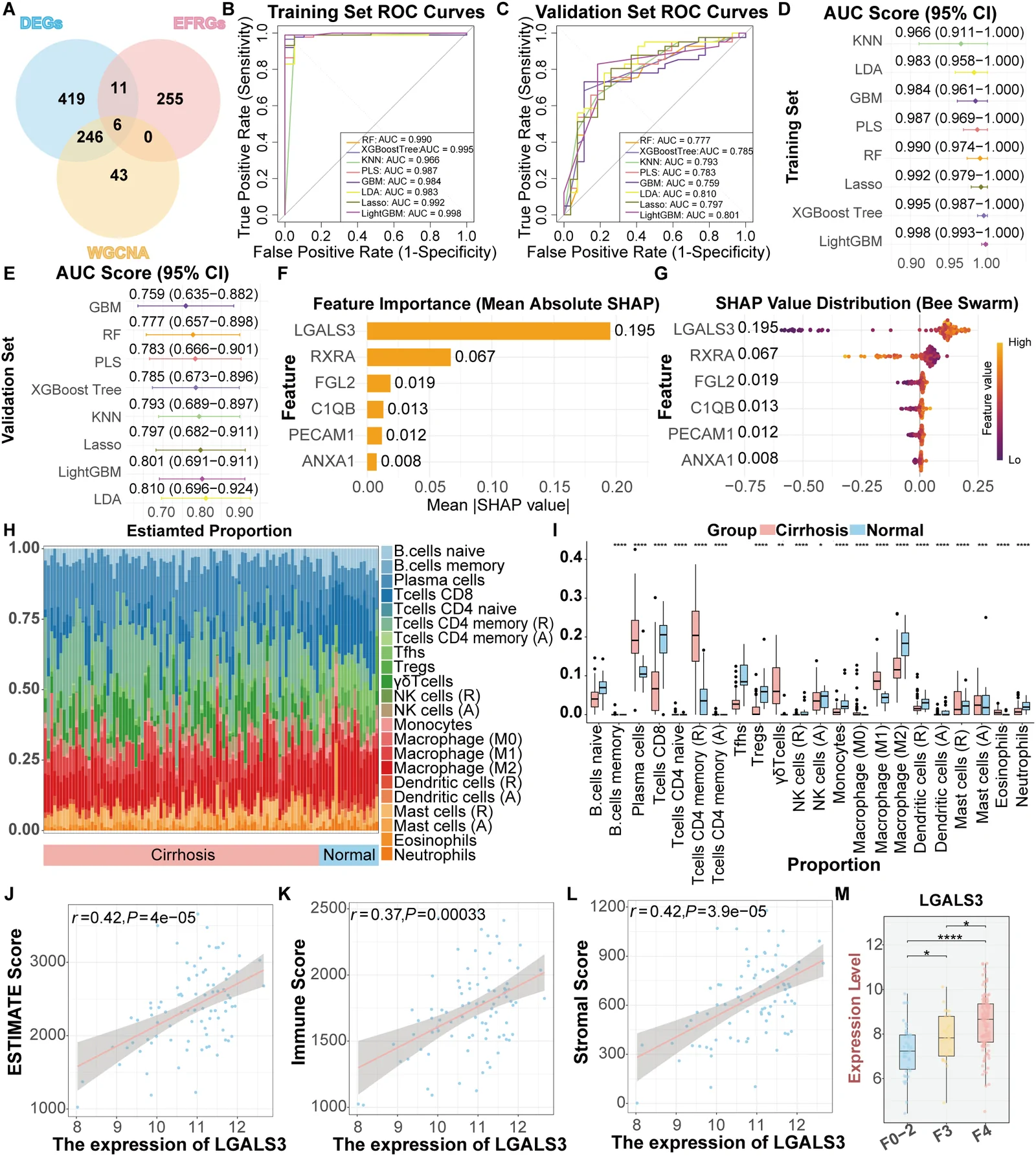
Identification of the efferocytosis-related hub gene and differences in immune infiltration between the normal and cirrhotic groups. **(A)** Venn diagram of the three groups: DEGs, EFRGs, and WGCNA. **(B, C)** ROC curves of the eight machine learning models in the training set **(B)** and validation set **(C)**. **(D, E)** Forest plots of the AUC scores of each model in the training set **(D)** and validation set **(E)**. **(F, G)** SHAP plots of the optimal model, including a feature importance bar chart **(F)** and a beeswarm plot **(G)**. **(H, I)** Stacked bar graph **(H)** and boxplot **(I)** of the differences in the proportions of immune cells in the normal and cirrhotic groups. **(J–L)** Correlation between LGALS3 expression and the **(J)** ESTIMATE score, **(K)** immune score, and **(L)** stromal score in the cirrhotic group. **(M)** Boxplot of LGALS3 expression in samples with different fibrosis stages. *P < 0.05; **P < 0.01; ***P < 0.001; and ****P < 0.0001.

### 3.4 LDA-based diagnostic model highlights LGALS3 as a key efferocytosis-related marker in LC

The performance of each of the 8 diagnostic models for predicting LC occurrence was evaluated on the basis of efferocytosis-related candidate gene expression data. LDA showed the best diagnostic performance, with a cross-validated AUC of 0.983 in the training set and 0.810 for the validation set (95% CI: 0.958-1.000 and 0.696-0.924, respectively) (**Figures 3B–E**), indicating that LDA was the optimal model for LC diagnosis. After the final LDA model was fitted to the complete training set, the apparent AUC estimated on the full training set was 0.994. Internal validation using 1, 000 stratified bootstrap resamples yielded a mean optimism of 0.006 and an optimism-corrected AUC of 0.988, with all 1, 000 resamples completed successfully (**Supplementary Table S12**). Through SHAP analysis, we determined the contribution levels of each gene to the prediction of this optimal model. LGALS3 presented the highest mean absolute SHAP value (**Figure 3F**). Moreover, high LGALS3 expression significantly increased the model’s probability of predicting samples as LC (**Figure 3G**).

### 3.5 LGALS3 expression is associated with immune microenvironment alterations in LC

The proportion of inflammatory macrophages was significantly greater in the LC group, whereas the percentage of non-inflammatory macrophages was markedly lower (t test, *P* < 0.0001) (**Figures 3H, I**), indicating an imbalance in the proportions of these two types of macrophages in the context of LC. This finding provided a strong rationale for our subsequent research. ESTIMATE algorithm analysis revealed that all three scores were significantly higher in cirrhotic samples than in normal samples (t test, *P* < 0.001) (**Supplementary Figure S2C**). In the LC samples, LGALS3 expression was positively correlated with all three scores, as determined by Spearman correlation analysis (ESTIMATE score: r = 0.42, *P* = 4.0 × 10^-5^; Immune score: r = 0.37, *P* = 3.3 × 10^-4^; and Stromal score: r = 0.42, *P* = 3.9 × 10^-5^) (**Figures 3J–L**).

### 3.6 LGALS3 expression gradually increases during liver fibrosis progression and is higher in cirrhotic liver tissues

LGALS3 expression increased with increasing fibrosis severity (Wilcoxon rank-sum test, *P* < 0.0001) (**Figure 3M**). H&E staining revealed that compared with those from the respective control groups, both human cirrhotic liver tissues and liver tissues from BDL mice exhibited marked disruption of the hepatic lobular architecture, increased inflammatory cell infiltration, and varying degrees of fibrous septum formation. Masson staining and Sirius Red staining demonstrated significantly increased collagen fibre deposition in both human cirrhotic liver tissues and BDL mouse liver tissues (**Figure 4A**). Quantitative analyses of Masson and Sirius Red staining intensities demonstrated increased collagen deposition in cirrhotic liver tissues (t test, *P* < 0.001) (**Figures 4B–E**). To verify the association between LGALS3 and LC, IHC and IF staining of LGALS3 were performed, and the results revealed that LGALS3 expression was significantly increased in both human cirrhotic liver tissues and BDL mouse liver tissues (t test, *P* < 0.001) (**Figures 4F–M**). WB analysis revealed similar results (t test, *P* < 0.001) (**Figures 4N–P**).

**Figure 4:**
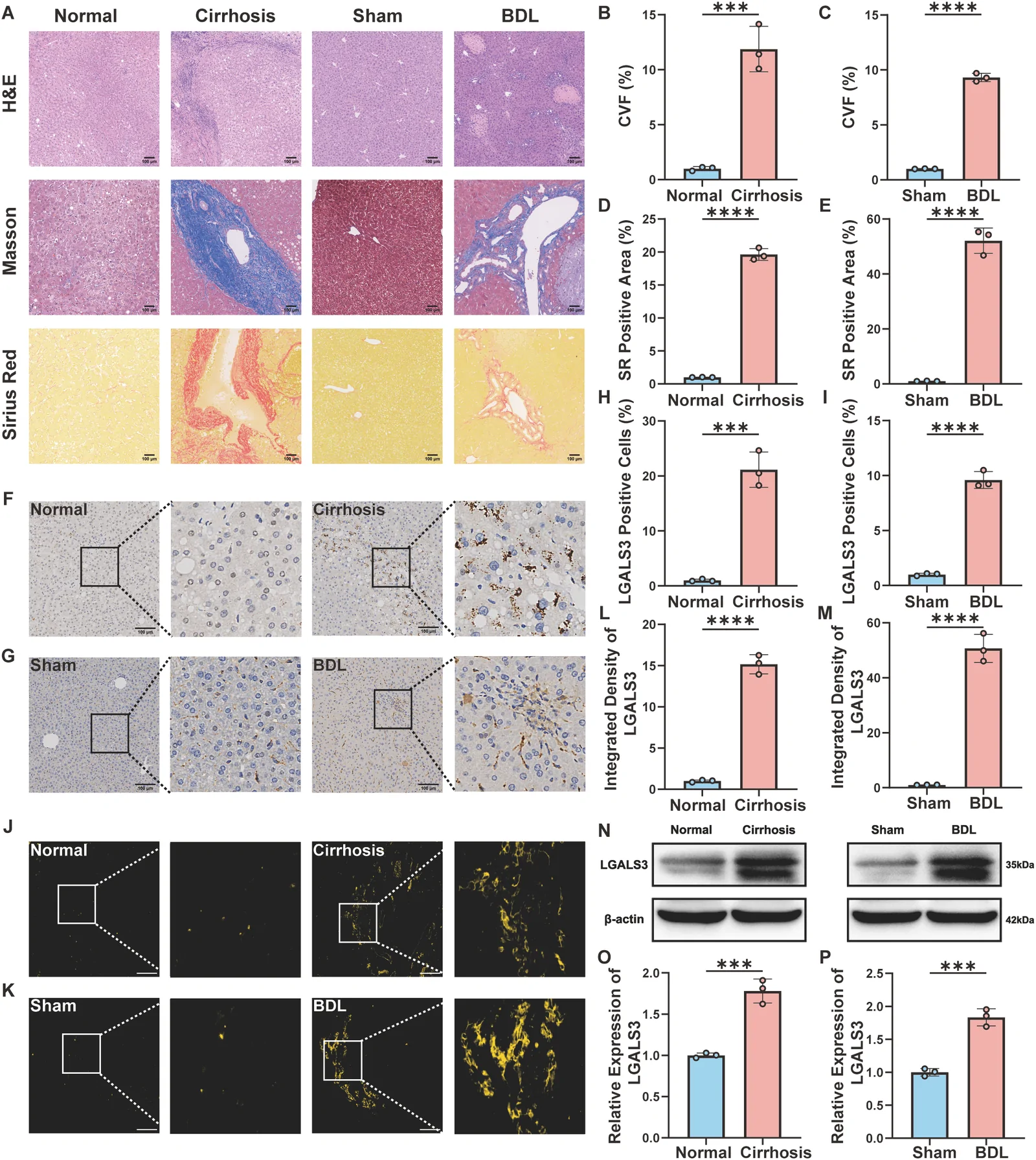
Verification of histopathological changes and changes in LGALS3 expression in human and mouse liver tissues. **(A)** Representative images of H&E, Masson, and Sirius Red staining of normal and cirrhotic liver tissues and sham and BDL mouse liver tissues (scale bars = 100 μm). **(B, C)** Comparison of the collagen volume fraction (CVF) between the normal and cirrhotic groups **(B)** and between the sham and BDL groups **(C)**. **(D, E)** Comparison of the Sirius Red-positive areas between the normal and cirrhotic groups **(D)** and between the sham and BDL groups **(E)**. **(F, G)** Representative IHC images of LGALS3 in normal and cirrhotic liver tissues **(F)** and sham and BDL mouse liver tissues **(G)** (scale bars = 100 μm). **(H, I)** Percentage of LGALS3^+^ cells in the normal and cirrhotic groups **(H)** and in the sham and BDL groups **(I)**. **(J, K)** Representative IF images of LGALS3 in normal and cirrhotic liver tissues **(J)** and sham and BDL mouse liver tissues **(K)** (scale bars = 100 μm). **(L, M)** Integrated density of LGALS3 in the normal and cirrhotic groups **(L)** and in the sham and BDL groups **(M)**. **(N)** Representative WB images of LGALS3 and β-actin expression in normal and cirrhotic liver tissues and sham and BDL mouse liver tissues. **(O, P)** Quantitative analysis of LGALS3 protein expression normalized to that of β-actin in the normal and cirrhotic groups **(O)** and in the sham and BDL groups **(P)**. ***P < 0.001 and ****P < 0.0001.

### 3.7 LGALS3 is highly expressed in cirrhotic inflammatory macrophages

Cells were partitioned into 11 distinct clusters (**Figure 5A**). LGALS3 expression across all the cell populations, as shown in the UMAP feature plot (**Figure 5D**), differential expression volcano plot (log_2_ (FC) =1.544, *P* < 0.001) (**Supplementary Figure S2D**; **Supplementary Table S13**) and violin plot (**Supplementary Figure S2E**), was highest in MPs. Moreover, LGALS3 expression in cirrhotic MPs was notably greater than that in normal macrophages (Wilcoxon rank-sum test, *P* < 0.001) (**Figure 5E**).

**Figure 5:**
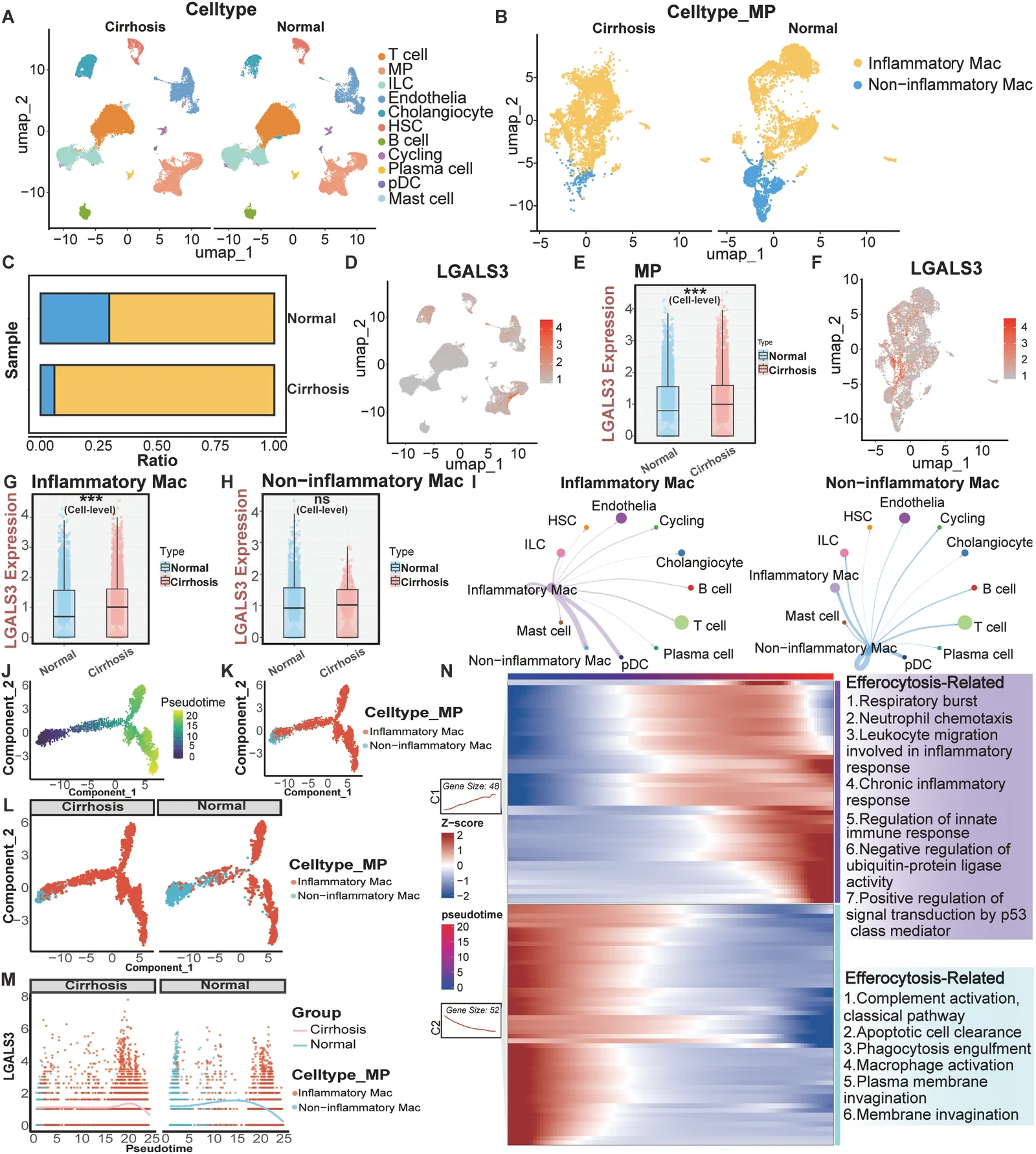
LGALS3 expression and pseudotime analysis of MP subpopulations. **(A, B)** UMAP plot of the cell population distribution **(A)** and MP subpopulation distribution **(B)** in the normal and cirrhotic groups. **(C)** Proportions of MP subpopulations in the normal and cirrhotic groups. **(D)** UMAP plot of the distribution of LGALS3 expression in the cell populations. **(E)** Comparison of LGALS3 expression in MPs between the normal and cirrhotic groups at the cell level. The corresponding donor-level analysis is shown in **Supplementary Figure S3A**. **(F)** UMAP plot of the distribution of LGALS3 expression in MPs. **(G, H)** Comparison of LGALS3 expression in inflammatory macrophages **(G)** and non-inflammatory macrophages **(H)** between the normal and cirrhotic groups at the cell level. The corresponding donor-level analysis is shown in **Supplementary Figure S3B**. **(I)** Interaction strength of non-inflammatory and inflammatory macrophages with other cell populations in the cirrhotic group. **(J)** Two-dimensional scatterplot of pseudotime. **(K)** Two-dimensional scatterplot of the cell trajectory from non-inflammatory macrophages to inflammatory macrophages. **(L)** Two-dimensional scatterplot of the cell trajectory from non-inflammatory macrophages to inflammatory macrophages in the normal and cirrhotic groups. **(M)** Plot of LGALS3 expression in the cell trajectory from non-inflammatory macrophages to inflammatory macrophages in the normal and cirrhotic groups. **(N)** Heatmap and enrichment analysis of pseudotime-associated differentially expressed genes from non-inflammatory macrophages to inflammatory macrophages. ***P < 0.001. “ns” means “not significant” (P ≥ 0.05).

We further conducted a refined annotation of MP subpopulations and classified them as inflammatory or non-inflammatory macrophages (**Figure 5B**). The proportion of inflammatory macrophages among cirrhotic MPs was significantly greater than that among normal MPs (**Figure 5C**). LGALS3 was highly expressed mainly in inflammatory macrophages (**Figure 5F**). With respect to inflammatory macrophages, LGALS3 expression was significantly greater in cirrhotic inflammatory macrophages than in normal inflammatory macrophages (Wilcoxon rank-sum test, *P* < 0.001) (**Figure 5G**), whereas no such change in LGALS3 expression was detected in non-inflammatory macrophages (**Figure 5H**). After donor-level aggregation, the comparisons between normal and cirrhotic donors did not reach statistical significance (Wilcoxon rank-sum test) (**Supplementary Figures S3A, B**).

### 3.8 High LGALS3 expression may be associated with the transition of MPs from a non-inflammatory state to an inflammatory state

Close intercellular communication between inflammatory and non-inflammatory macrophages was detected in LC tissues (**Figure 5I**), suggesting potential functional coupling and dynamic state transitions between these two populations. Therefore, pseudotime analysis was further performed to explore their potential state-transition trajectory. The results revealed that non-inflammatory macrophages were located at the early stage of the trajectory, whereas inflammatory macrophages were distributed mainly at the late stage, suggesting a dynamic transition from a non-inflammatory state to an inflammatory state (**Figures 5J, K**). Both cirrhotic and normal MPs exhibited similar trends (**Figure 5L**). The peak expression of LGALS3 in cirrhotic MPs coincided with the timing of inflammatory macrophage accumulation, and no such variation was detected in normal MPs (**Figure 5M**). Additionally, we found that numerous efferocytosis-related pathways were enriched, with activation of the chronic inflammatory response pathway and suppression of the apoptotic cell clearance pathway (**Figure 5N**).

### 3.9 Comparison of efferocytic capacity between inflammatory and non-inflammatory macrophages

Among all the cell types tested, the efferocytic activity of the MPs was greatest (**Figure 6A**). Compared with normal MPs, cirrhotic MPs exhibited reduced efferocytic activity (t test, *P* < 0.05) (**Figure 6B**). After aggregation at the donor level, the comparisons between healthy and cirrhotic donors did not reach statistical significance (Wilcoxon rank-sum test) (**Supplementary Figure S3C**). Additionally, double IF staining for F4/80 and TUNEL revealed a significantly lower percentage of F4/80^+^TUNEL^+^ cells among the total TUNEL^+^ cell population in the BDL group than in the sham group (t test, *P* < 0.001) (**Figures 7I, J**). Meanwhile, total TUNEL-positive cell counts did not differ significantly between the sham and BDL groups (n = 3 per group, *P* = 0.2603; **Supplementary Table S14**). Together, these indirect measures were consistent with reduced efferocytic capacity in cirrhotic MPs. In addition, compared with non-inflammatory macrophages, inflammatory macrophages exhibit lower efferocytic activity in LC. Moreover, macrophages with high LGALS3 expression exhibited increased ECM deposition in LC (Wilcoxon rank-sum test, *P* < 0.001) (**Figures 6C, D**). Consistent trends were also observed in the corresponding donor-level paired analyses (paired *t* test, *P* < 0.01) (**Supplementary Figure S3D, E**). In cirrhotic MPs, inflammatory macrophages largely overlapped with those with low efferocytic capacity, whereas non-inflammatory macrophages overlapped with those exhibiting high efferocytic activity (**Figures 6E, F**). To assess whether the inclusion of LGALS3 in the efferocytosis-related gene set influenced the scRNA-seq analysis results, we repeated the AUCell and Scissor analyses after excluding LGALS3 from the gene set. The results were consistent with those of the original analyses, indicating that the inclusion of LGALS3 did not materially affect the main findings (panel G, *t* test; panel H, Wilcoxon rank-sum test; *P* < 0.001) (**Supplementary Figure S3F-J**). The results of the pseudotime analysis of MPs with high and low efferocytic capacity were highly consistent with those of the previous pseudotime analysis of non-inflammatory and inflammatory macrophages (**Figures 6G–K**).

**Figure 6:**
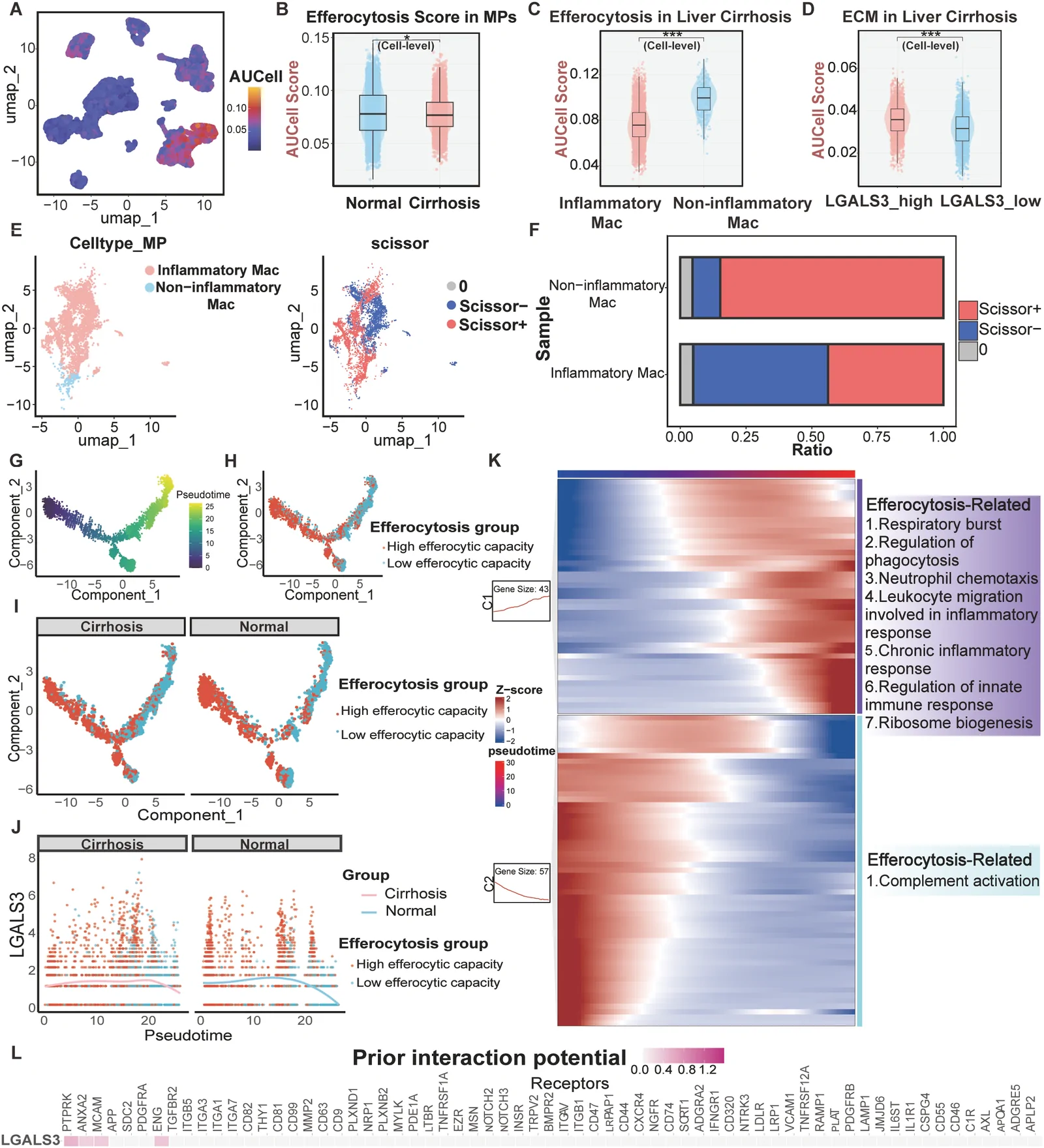
Single-cell analysis of efferocytic activity and pseudotime analysis of MPs with high or low efferocytic capacity. **(A)** AUCell score distribution UMAP plot of the efferocytosis signalling pathway. **(B)** Comparison of efferocytic activity in MPs between the normal and cirrhotic groups at the cell level. The corresponding donor-level analysis is shown in **Supplementary Figure S3C**. **(C)** Comparison of efferocytic activity between inflammatory and non-inflammatory macrophages in the cirrhotic group at the cell level. The corresponding donor-level analysis is shown in **Supplementary Figure S3D**. **(D)** Comparison of ECM deposition between LGALS3-high and LGALS3-low macrophages in the cirrhotic group at the cell level. The corresponding donor-level analysis is shown in **Supplementary Figure S3E**. **(E)** UMAP plot of the scissor analysis in the cirrhotic group. **(F)** Cell proportion diagram of the scissor analysis in inflammatory and non-inflammatory macrophages in the cirrhotic group. **(G)** Two-dimensional scatterplot of pseudotime. **(H)** Two-dimensional scatterplot of the cell trajectory from high efferocytic capacity to low efferocytic capacity macrophages. **(I)** Two-dimensional scatterplot of the cell trajectory from high efferocytic capacity to low efferocytic capacity macrophages in the normal and cirrhotic groups. **(J)** Plot of LGALS3 expression from high efferocytic capacity to low efferocytic capacity macrophages in the normal and cirrhotic groups. **(K)** Heatmap and enrichment analysis of pseudotime-associated differentially expressed genes from high efferocytic capacity to low efferocytic capacity macrophages. **(L)** The potential candidate receptors determined through NicheNet analysis between MPs and HSCs. *P < 0.05; ***P < 0.001.

**Figure 7:**
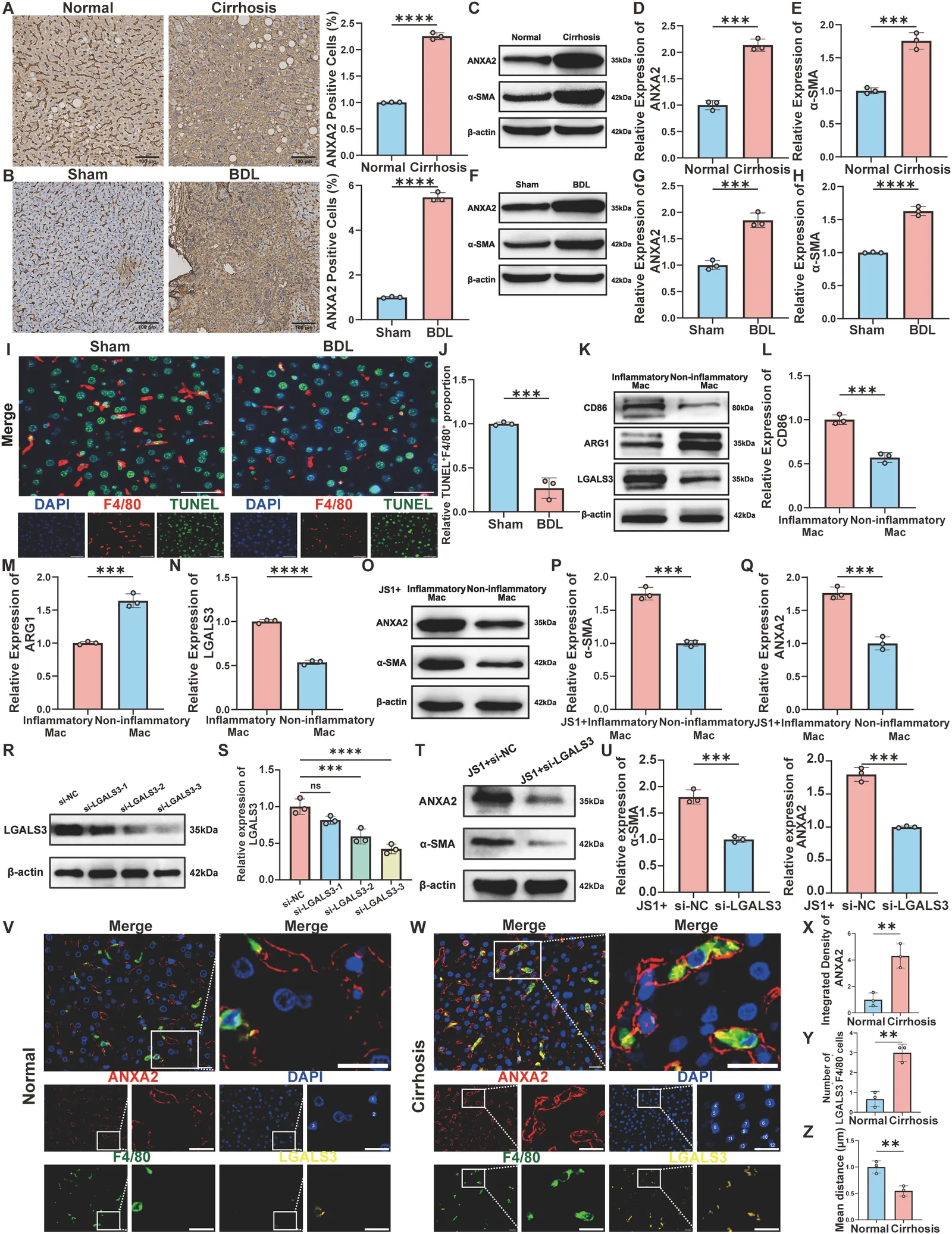
Experimental validation of ANXA2 expression and inflammatory macrophage-HSC communication via LGALS3-ANXA2. **(A, B)** Representative IHC images and percentages of ANXA2^+^ cells in human normal and cirrhotic liver tissues **(A)** and in liver tissues from sham and BDL mice **(B)** (scale bars = 100 μm). **(C–H)** Representative WB images and quantitative analysis of ANXA2 and α-SMA protein expression normalized to that of β-actin in normal and cirrhotic human liver tissues **(C–E)** and sham and BDL mouse liver tissues **(F–I)** Representative IF images of F4/80 (red) and TUNEL (green) in liver tissues from sham and BDL mice (scale bars = 50 μm). **(J)** Comparison of the proportion of TUNEL^+^ cells that were also F4/80-positive between the sham and BDL groups. The proportion was calculated as the number of TUNEL^+^F4/80^+^ cells divided by the total number of TUNEL^+^ cells and normalized to the sham group. **(K)** Representative WB images of CD86, ARG1, LGALS3 and β-actin expression in inflammatory and non-inflammatory macrophages. **(L–N)** Quantitative analysis of CD86 **(L)**, ARG1 **(M)**, and LGALS3 **(N)** protein expression normalized to that of β-actin in inflammatory and non-inflammatory macrophages. **(O)** Representative WB images of ANXA2, α-SMA and β-actin expression in JS1 cells after Transwell co-culture with inflammatory or non-inflammatory macrophages. **(P, Q)** Quantitative analysis of α-SMA **(P)** and ANXA2 **(Q)** protein expression normalized to that of β-actin in JS1 cells after Transwell co-culture with inflammatory or non-inflammatory macrophages. **(R)** Representative WB images of LGALS3 expression in inflammatory macrophages transfected with si-NC or the three different LGALS3-targeting siRNAs. **(S)** Quantitative analysis of LGALS3 protein expression normalized to that of β-actin in the four transfection groups. **(T)** Representative WB images of α-SMA and ANXA2 expression in JS1 cells co-cultured with inflammatory macrophages transfected with si-NC or the selected si-LGALS3 sequence. **(U)** Results from the quantitative analysis of α-SMA and ANXA2 protein expression normalized to that of β-actin in JS1 cells after Transwell co-culture with inflammatory macrophages transfected with si-NC or the selected si-LGALS3 sequence. **(V, W)** Representative IF images of ANXA2 (red), F4/80 (green), and LGALS3 (yellow) in normal **(V)** and cirrhotic **(W)** liver tissues (scale bars = 20 μm). **(X)** Integrated density of ANXA2 in the normal and cirrhotic groups. **(Y)** Comparison of the numbers of LGALS3^+^ F4/80^+^ cells between the normal and cirrhotic groups. **(Z)** Comparison of the mean distances between LGALS3^+^ F4/80^+^ cells and ANXA2^+^ cells in the normal and cirrhotic groups. **P < 0.01; ***P < 0.001; and ****P < 0.0001. “ns” means “not significant” (P ≥ 0.05).

### 3.10 Inflammatory macrophages may activate HSCs by binding to the candidate ANXA2 receptor on HSCs via the ligand LGALS3

Compared with normal samples, inflammatory macrophages, non-inflammatory macrophages, and HSCs exhibited stronger interactions and greater number of communications in cirrhotic samples (**Supplementary Figures S2F, G**). NicheNet analysis predicted ANXA2, PTPRK, ENG, and MCAM as four potential candidate receptors on HSCs (**Figure 6L**). PRODIGY-estimated binding energies of LGALS3 with all four candidate receptors were less than −7 kcal/mol; however, the AlphaFold-predicted complexes showed pTM values ranging from 0.42 to 0.56 and ipTM values ranging from 0.11 to 0.44 (**Supplementary Table S15**). The relatively low ipTM scores indicated limited confidence in the predicted interprotein interfaces. Therefore, these structural predictions and the PRODIGY-derived binding free energies were considered exploratory and were not used to discriminate among the candidate receptors (**Figures 8A–D**). Comparison of the expression patterns of the four candidate receptors showed that ANXA2 was the only receptor that both increased with fibrosis stage in bulk transcriptomic data and was specifically upregulated in HSCs from cirrhotic livers. Although MCAM showed a stage-related increase in bulk data, its expression was decreased in cirrhotic HSCs at the single-cell level, whereas ENG and PTPRK showed no clear stage-related changes. Therefore, ANXA2 was prioritised for subsequent validation based on its fibrosis-associated and HSC-specific expression pattern (**Supplementary Figures S4A-F**). During the final 50 ns of the molecular dynamics simulation, the root mean square deviation (RMSD) of the LGALS3–ANXA2 complex showed limited fluctuation, with a mean value of 5.60 ± 0.21 Å and a range of 4.84–6.16 Å (**Figure 8E**). Most of the root mean square fluctuation (RMSF) values were less than 5 Å (**Figure 8F**). Moreover, during the analyzed trajectory, the LGALS3–ANXA2 complex maintained an average of 2.17 ± 1.34 interfacial hydrogen bonds. Notably, the interfacial hydrogen-bond count was relatively low and showed substantial variability throughout the trajectory. (**Figure 8G**). Visual inspection of the trajectory showed that the LGALS3–ANXA2 complex remained associated during the 100-ns simulation. (**Figure 8H**). During the 100-ns molecular dynamics simulation, the predicted LGALS3–ANXA2 complex remained associated throughout the simulated trajectory. However, the continued increase in the ANXA2 RMSD toward the end of the simulation indicated incomplete convergence, and a single simulation replicate was insufficient to establish binding. IHC analysis demonstrated higher ANXA2 expression in human cirrhotic livers and BDL mouse livers than in their respective control tissues (t test, *P* < 0.0001) (**Figures 7A, B**). WB demonstrated consistent findings, especially for ANXA2 and α-SMA (a marker of activated HSCs) (t test, *P* < 0.001) (**Figures 7C–H**) (21). These findings were similar to the previously detected trend in LGALS3 expression.

**Figure 8:**
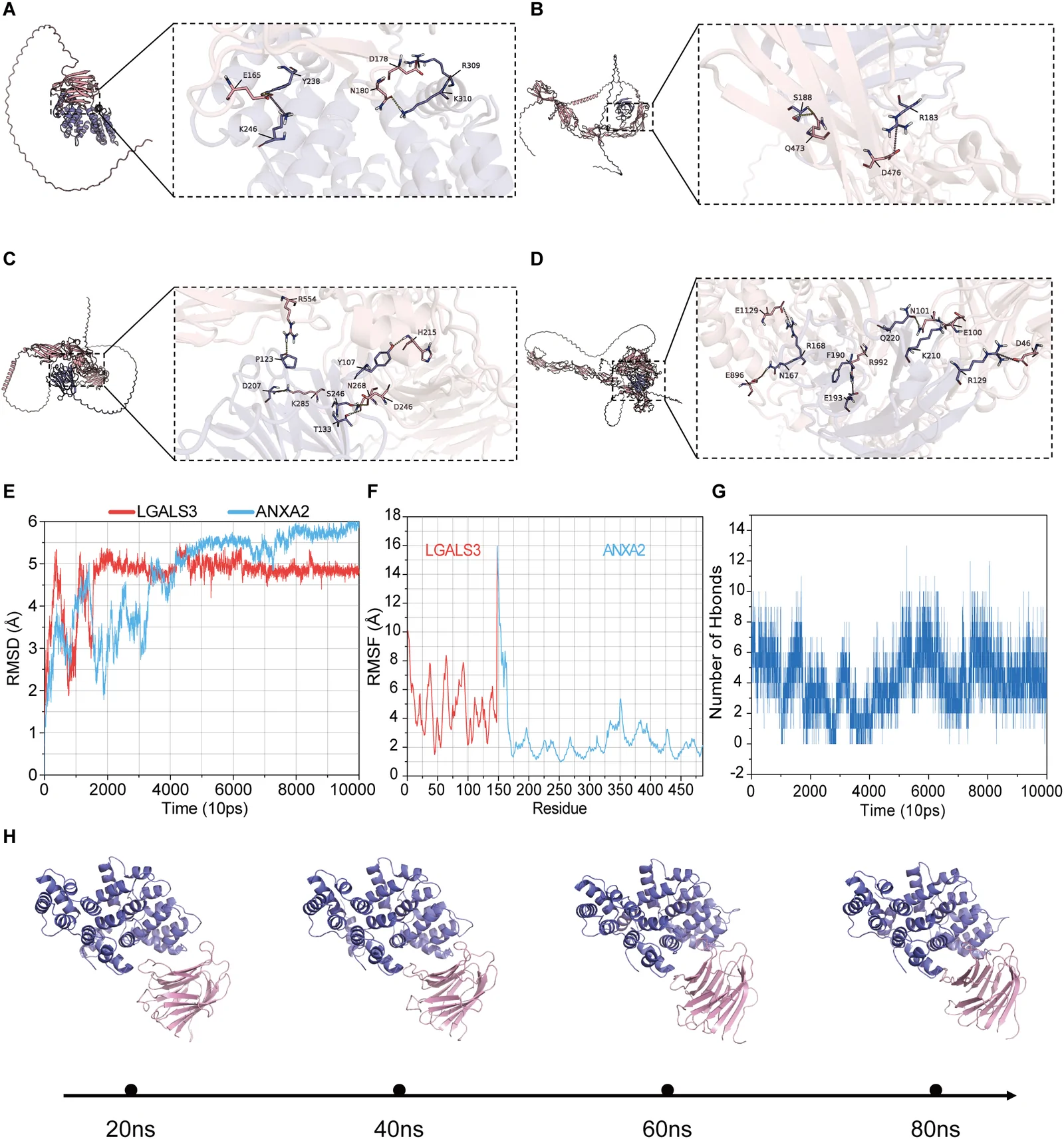
AlphaFold complex structure prediction and molecular dynamics simulations. **(A)** AlphaFold complex structure prediction between LGALS3 and ANXA2. **(B)** AlphaFold complex structure prediction between LGALS3 and MCAM. **(C)** AlphaFold complex structure prediction between LGALS3 and ENG. **(D)** AlphaFold complex structure prediction between LGALS3 and PTPRK. **(E–G)** Molecular dynamics simulations of the LGALS3-ANXA2 complex, including the RMSD **(E)**, RMSF **(F)**, and number of hydrogen bonds **(G)**. **(H)** Diagram of the conformational changes at different time points during the molecular dynamics simulations.

These findings were then validated at the cellular level. WB analysis revealed that CD86 expression was markedly upregulated in inflammatory macrophages, whereas ARG1 expression was markedly upregulated in non-inflammatory macrophages (t test, *P* < 0.001), confirming successful macrophage polarization. LGALS3 expression was significantly greater in inflammatory macrophages than in non-inflammatory macrophages (t test, *P* < 0.0001) (**Figures 7K–N**). These findings were concordant with the scRNA-seq results. To further examine the effect of LGALS3 derived from inflammatory macrophages on HSC activation, we used a Transwell co-culture system. Compared with those in the non-inflammatory macrophage-JS1 co-culture group, the expression levels of ANXA2 and α-SMA in JS1 cells were markedly increased in the inflammatory macrophage-JS1 co-culture group (t test, *P* < 0.001) (**Figures 7O–Q**). Among the three si-LGALS3 sequences tested, si-LGALS3–3 exhibited the highest knockdown efficiency and was therefore selected for subsequent experiments (one-way ANOVA, P < 0.0001) (**Figures 7R, S**). ANXA2 and α-SMA levels in JS1 cells were significantly lower after co-culture with si-LGALS3-transfected than with si-NC-transfected inflammatory macrophages (t test, P < 0.001) (**Figures 7T, U**). IF co-localization and spatial distance analyses demonstrated that the total fluorescence intensity of ANXA2 was markedly greater in cirrhotic liver tissues than in normal liver tissues, indicating a pattern similar to that of LGALS3. Moreover, the number of LGALS3^+^ F4/80^+^ cells significantly increased in cirrhotic liver tissues, indicating a marked increase in the number of LGALS3+ macrophages. Further analysis revealed that, the spatial distance between LGALS3^+^ macrophages and ANXA2^+^ cells was significantly smaller in cirrhotic liver tissues than in normal liver tissues (t test, *P* < 0.01) (**Figures 7V–Z**). These results indicate a closer spatial association between the two cell types and support the possibility of a local interaction. These findings suggested that inflammatory macrophages might promote HSC activation through an LGALS3–ANXA2 communication axis.

## 4 Discussion

The global burden of LC is severe, and current medical interventions offer limited survival benefits for patients with advanced disease (1). Therefore, the identification of reliable targets for early diagnosis and therapeutic intervention in LC is of critical importance. Previous evidence has shown that impaired macrophage efferocytosis delays the resolution of liver fibrosis, suggesting that weakened efferocytic capacity may contribute to cirrhosis progression (22). Further studies are needed to define the specific mechanism involved (9).

We combined WGCNA, machine learning, and SHAP analysis to identify LGALS3 as a candidate efferocytosis-related biomarker for the diagnosis of LC. To validate whether LGALS3 and its potential downstream alterations persist in cirrhotic tissues, we performed experimental validation using both human cirrhotic liver samples and the BDL model. Although the BDL model cannot fully recapitulate the heterogeneous etiologies and chronic pathological processes of human cirrhosis (23, 24), it is a well-established and widely used experimental model of cholestasis-induced liver injury and secondary biliary cirrhosis. Previous studies have shown that BDL is widely used to model cholestasis-induced liver injury and secondary biliary cirrhosis, recapitulating key pathological features such as inflammation, ductular reactions, HSC activation, collagen deposition, and progressive fibrosis/cirrhosis (25–28). On the basis of an analysis of public databases and the results of IHC, WB, and IF analyses of human and mouse liver tissue samples, we found that LGALS3 expression increased in parallel with fibrosis progression and that the expression levels of LGALS3 in cirrhotic samples were significantly greater than those in normal samples. These findings collectively identified LGALS3 as a candidate efferocytosis-related biomarker in LC.Galectins are β-galactoside-binding proteins characterized by a conserved carbohydrate-recognition domain (CRD) comprising approximately 130 amino acids. Fifteen mammalian galectins have been reported and are categorized into three structural groups. LGALS3 is the only chimaera-type galectin and is characterized by a C-terminal CRD and a large N-terminal protein-interacting domain (29). LGALS3 is associated with a broad range of biological activities and has been reported to have notable roles in inflammatory regulation and immune cell recruitment (30), cell adhesion and migration (31), and cell growth and apoptosis (32). LGALS3 not only promotes fibrosis by facilitating macrophage polarization and activating HSCs to stimulate collagen fibre deposition but also acts as a bridge linking immune inflammation and LC (33, 34). Notably, LGALS3 has already been pursued as a therapeutic target in liver disease, with the LGALS3 inhibitor belapectin (GR-MD-02) having undergone clinical evaluation in patients with NASH/MASH-related advanced fibrosis or cirrhosis and portal hypertension, including the recent NAVIGATE trial evaluating the prevention of oesophageal varices (35–37). This clinical development programme is consistent with our findings. However, the specific macrophage subpopulation targeted by LGALS3 in LC tissue and how it mediates macrophage-HSC interactions to drive disease progression remain to be clarified. On the basis of these data, our study expanded the understanding of how LGALS3 promotes the progression of LC by examining two aspects: impaired efferocytic capacity in macrophages and intercellular communication between macrophages and HSCs.

Our findings revealed that LGALS3 was predominantly expressed in macrophages and that its expression was further increased in cirrhotic macrophages, suggesting a potential role for LGALS3 in macrophage dysfunction during cirrhosis progression (38). Notably, both scRNA-seq AUCell analysis and efferocytosis assessment based on TUNEL^+^F4/80^+^ cells consistently revealed that efferocytic activity was reduced in cirrhotic macrophages. After donor-level aggregation, the comparisons between normal and cirrhotic donors did not reach statistical significance, except for the paired t-test comparisons of mean efferocytosis and ECM activity AUCell scores among cirrhotic donors. These null results should be interpreted in the context of the limited number of donors and do not necessarily contradict the principal single-cell observations, which primarily concern the direction and topology of macrophage state transitions rather than the magnitude of any single between-group contrast. Accordingly, the lack of donor-level statistical significance does not alter the overall interpretation of these single-cell findings. Efferocytosis by hepatic macrophages has been reported to decrease markedly as liver fibrosis progresses (39). Moreover, after macrophages were subdivided into inflammatory and non-inflammatory subpopulations, integrated scissor analysis further revealed that inflammatory macrophages exhibited weaker efferocytic activity than non-inflammatory macrophages did (40). These results are biologically plausible because IL-4 and efferocytosis additively promote the proliferation of non-inflammatory macrophages, whereas IFN-γ/LPS-induced inflammatory macrophages display a markedly blunted response to efferocytosis (16, 17). Pseudotime analysis (41) revealed that LGALS3 expression peaked as macrophages shifted towards an inflammatory phenotype with reduced efferocytic activity. These findings revealed that LGALS3 marks a pro-inflammatory, low efferocytic capacity hepatic macrophage state. Notably, the inflammatory macrophage compartment identified in our analysis is closely related to the TREM2^+^CD9^+^ scar-associated macrophage population described by Ramachandran et al., a profibrotic macrophage population in which LGALS3 is an established marker (15). This connection places our findings within the established scar-associated macrophage framework and further suggests that LGALS3 may participate in the profibrotic communication between inflammatory macrophages and HSCs.

Accumulating evidence indicates that hepatic stellate cell activation is orchestrated by multiple, complex molecular regulatory mechanisms (42, 43). LGALS3 plays an important role in HSC activation. Henderson et al. reported that LGALS3 plays a key role in myofibroblast activation and procollagen deposition. LGALS3 is essential for TGF-β-mediated myofibroblast activation and collagen deposition (44). Jiang et al. reported that LGALS3 induces the expression of TGF-β1 and collagen-related molecules through the integrin αvβ3-dependent phagocytosis of apoptotic cells by HSCs and by further amplifying HSC activation via NF-κB-mediated positive feedback (45). In contrast, the precise molecular mechanism through which LGALS3 mediates inflammatory macrophage-HSC communication and promotes HSC activation in the cirrhotic microenvironment remains unclear. NicheNet analysis was used to identify the receptor on HSCs through which MP-derived LGALS3 may promote the transition of HSCs from a healthy state to a cirrhotic state (46). A total of four candidate receptors, namely, ANXA2, PTPRK, ENG and MCAM, were predicted to be potential candidate receptors. Notably, all four candidate LGALS3–receptor complexes showed predicted binding energies below −7 kcal/mol, indicating that the molecular modelling did not discriminate among the four candidates and therefore was not used to prioritize ANXA2.ANXA2 belongs to the vertebrate annexin family, a group of calcium-dependent anionic phospholipid-binding proteins. ANXA2 is widely expressed in endothelial cells, monocytes, macrophages, and various cancer cells and may exist as either a monomer or an S100A10-associated heterotetramer. ANXA2 performs a range of biological functions both at the cell surface and intracellularly, including fibrinolysis, angiogenesis, and cancer progression (47, 48). ANXA2 expression has been reported to be elevated in fibrosis-related liver diseases and is closely associated with HSC activation and collagen deposition. In addition, ANXA2 can promote STAT3 phosphorylation and its downstream transcriptional activity, suggesting that ANXA2 may participate in HSC activation through a STAT3-related signalling pathway (49, 50). ENG is closely associated with HSC activation and TGF-β signalling (51). However, HSC-specific deletion of ENG has been shown to aggravate liver fibrosis in experimental models (52), suggesting that the role of ENG in HSC activation and fibrogenesis is complex and context dependent rather than uniformly profibrogenic. With respect to PTPRK, recent liver-related studies have focused mainly on the role of this factor in hepatocyte metabolic reprogramming and hepatocarcinogenesis rather than HSC activation or liver fibrosis (53). Similarly, MCAM (CD146) is used primarily as a marker of liver sinusoidal endothelial cells and other vascular-associated cells, but direct evidence that it promotes HSC activation or collagen production remains limited (54). Further analysis of high-throughput transcriptomic data during the progression of liver fibrosis and scRNA-seq data revealed that ANXA2 and MCAM expression generally increased as fibrosis advanced and was significantly greater at the late stage than at the early stage, whereas no comparable changes were detected for ENG or PTPRK. Moreover, ANXA2 expression was significantly elevated in cirrhotic HSCs compared with that in normal HSCs, whereas MCAM expression exhibited the opposite pattern. Based on these findings, ANXA2 was the only candidate that both increased with fibrosis stage and was specifically elevated in cirrhotic HSCs, so ANXA2 was selected as the receptor of interest for subsequent validation.

After identifying ANXA2 as the candidate receptor, we integrated molecular dynamics simulations, tissue expression analyses, spatial proximity assessments, and cellular functional experiments to systematically support the potential role of the LGALS3-ANXA2 axis in HSC activation during LC progression. Molecular dynamics simulations revealed that the conformation of the LGALS3-ANXA2 complex remained associated throughout the simulations. At the tissue level, ANXA2 expression was markedly increased in both human cirrhotic liver tissues and BDL mouse liver tissues, with an expression pattern consistent with that of upregulated LGALS3. In addition, the number of LGALS3^+^ macrophages was greater in human cirrhotic liver tissues than in normal tissues. Moreover, in the fibrotic septa-adjacent regions of human cirrhotic liver tissues, the intercellular distance between LGALS3^+^ macrophages and ANXA2^+^ cells was significantly smaller than that in normal liver tissues, indicating closer spatial proximity and supporting the possibility of local interactions. Furthermore, co-culture experiments demonstrated that inflammatory macrophages with high LGALS3 expression promoted HSC activation and increased α-SMA expression. LGALS3 knockdown in inflammatory macrophages markedly attenuated the upregulation of ANXA2 and α-SMA in co-cultured JS1 cells relative to si-NC, indicating that inflammatory macrophagederived LGALS3 is necessary for the full HSC response under these conditions.

Overall, the principal contribution of our study lies in placing LGALS3 within a defined macrophage-state and intercellular-communication framework in LC. By integrating scRNA-seq analysis with multilevel experimental validation, including IHC, IF, and WB, our major findings can be summarized into two key aspects. First, macrophages are primarily responsible for efferocytosis. LGALS3 was predominantly expressed by the inflammatory macrophage subpopulation. Further analyses suggested that LGALS3 marks a pro-inflammatory, low efferocytic capacity hepatic macrophage state.

Second, inflammatory macrophage-derived LGALS3 may interact with ANXA2 expressed on HSCs, thereby contributing to HSC activation. Previous studies have shown that ANXA2 may engage downstream STAT3 signalling, including STAT3 phosphorylation, dimerization, and nuclear translocation, thereby enhancing the transcription of the HSC activation-related gene α-SMA and ultimately driving HSC activation and fibrotic progression (49, 50). Accordingly, we present this literature-supported proposed working model in **Figure 9**.

**Figure 9:**
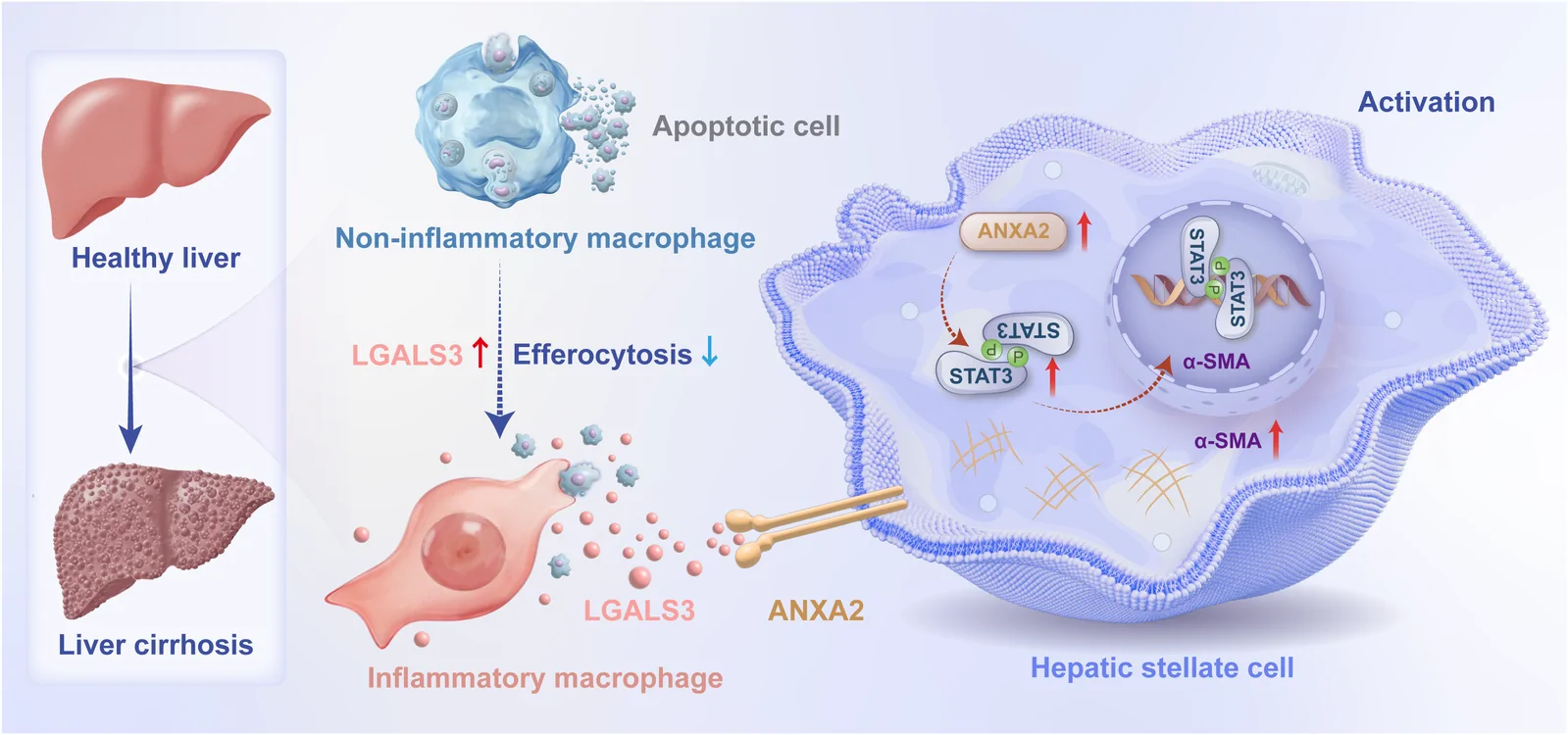
Proposed working model. Solid arrows indicate relationships directly supported by data in the present study. Dashed arrows indicate proposed or inferred relationships that were not directly demonstrated here, including the link between LGALS3 and reduced efferocytosis and the ANXA2–STAT3–α-SMA signalling module. The LGALS3–ANXA2 ligand–receptor engagement is predicted rather than experimentally demonstrated.

Despite the integration of bioinformatics analyses, scRNA-seq analysis, and multilevel experimental validation, several limitations of this study should be acknowledged. First, the limited sample size may affect the robustness of our conclusions and contribute to potential model overfitting, as reflected by the decrease in the cross-validated AUC from 0.983 in the training cohort to 0.810 in the validation cohort. Although bootstrap internal validation of the final six-gene LDA model indicated limited optimism, with an apparent AUC of 0.994 on the full training set, a mean optimism of 0.006, and an optimism-corrected AUC of 0.988, this result does not eliminate concerns regarding its external generalizability. In addition, cohort heterogeneity and differences in data distribution between the training and validation cohorts may have contributed substantially to this decline in external performance, particularly because both cohorts were constructed by merging multiple independent GEO series generated using different array platforms. Notably, GSE17967 contributed 47 cirrhotic samples but no normal controls to the merged training cohort, which may have increased cohort heterogeneity and introduced partial confounding between dataset origin and disease status, potentially contributing to the reduced performance observed in the independent validation cohort. Therefore, these findings and the generalizability of the diagnostic model require validation in larger, independent cohorts. Second, the cirrhotic liver tissues used for human validation were obtained from areas adjacent to HCC. Therefore, these peritumoural cirrhotic tissues may not fully represent cirrhotic liver tissue from patients without HCC, and a tumour-related field effect cannot be excluded. Future studies should validate these findings using cirrhotic liver tissues obtained from patients without concurrent HCC. Notably, the human validation cohort was also limited in composition, as all three cirrhotic donors were male, had HBV-related cirrhosis, and were classified as Child–Pugh B, whereas the control group comprised one male and two female donors. Third, although scRNA-seq analysis and the efferocytosis assessment based on TUNEL^+^F4/80^+^ cells consistently suggested reduced efferocytic activity in cirrhotic macrophages, particularly inflammatory macrophages, these approaches provide only indirect evidence rather than direct functional measurements. In addition, the proportion of TUNEL+ cells that are also F4/80+ may be influenced by changes in the overall abundance and cellular composition of apoptotic cells, including hepatocyte and cholangiocyte apoptosis, and therefore may not exclusively reflect macrophage efferocytic function. However, the total number of TUNEL+ cells did not differ significantly between the sham and BDL groups (*P* = 0.2603; **Supplementary Table S14**), arguing against a large denominator effect. Nevertheless, given the small sample size (n = 3 per group), more subtle differences in apoptotic cell abundance cannot be excluded. Moreover, the observed association between increased LGALS3 expression and the transition of macrophages toward an inflammatory state with impaired efferocytic capacity does not establish a direct regulatory role for LGALS3 in this process. Therefore, future studies should combine LGALS3 knockdown or overexpression with direct functional efferocytosis assays, such as flow cytometric quantification of macrophage uptake of pHrodo-labeled apoptotic cells, to confirm impaired efferocytic capacity in inflammatory macrophages from cirrhotic livers and determine whether LGALS3 directly regulates this process. Notably, the donor-level comparisons of the single-cell findings did not reach statistical significance except for the paired t-test comparisons of mean efferocytosis and ECM activity AUCell scores among cirrhotic donors; the limited number of donors therefore warrants caution when interpreting these observations. Fourth, although the LGALS3–ANXA2 complex remained associated throughout the 100-ns molecular dynamics trajectory, this finding reflects the behaviour of the selected initial model under the specified simulation conditions. Given the low ipTM score of the initial AlphaFold complex, the simulation does not independently validate the predicted interface or establish a direct physical interaction between LGALS3 and ANXA2. In future studies, surface plasmon resonance should be performed to comprehensively validate the direct binding affinity between LGALS3 and ANXA2 and strengthen the mechanistic evidence for the role of the LGALS3-ANXA2 axis in LC progression. Fifth, although LGALS3 knockdown in inflammatory macrophages markedly attenuated the upregulation of ANXA2 and α-SMA in co-cultured HSCs, this finding does not establish that the reduced HSC response resulted specifically from the loss of secreted LGALS3 signalling through ANXA2. Intracellular LGALS3 has been reported to act as an LPS sensor that promotes macrophage glycolysis through mTORC1 (18). Therefore, silencing LGALS3 in IFN-γ/LPS-polarised macrophages may alter the macrophage activation state and broader secretory profile, which could indirectly attenuate HSC activation. Moreover, we did not directly quantify secreted LGALS3 in the macrophage-conditioned medium. Thus, the extracellular release of LGALS3 and its specific contribution to macrophage–HSC communication remain to be confirmed. Future studies should assess macrophage activation markers and secretome changes following LGALS3 knockdown, measure LGALS3 levels in conditioned medium using ELISA or immunoblotting, and further evaluate the potential LGALS3–ANXA2 interaction using neutralising antibodies, recombinant LGALS3, or receptor-specific inhibition. Sixth, although three independent siRNA sequences targeting LGALS3 were initially screened, only the sequence with the highest knockdown efficiency was carried forward into the functional co-culture experiments. Therefore, a potential contribution from sequence-specific off-target effects cannot be formally excluded. Future studies should confirm these findings using additional independent siRNA sequences, rescue experiments, or alternative gene-silencing approaches. Furthermore, only male mice were included in the animal experiments, and potential sex-related differences were not evaluated. Seventh, the cellular experiments were conducted using the murine macrophage cell line RAW264.7 and the murine HSC line JS1, whereas the hypothesis was primarily generated from human scRNA-seq data. Further validation using primary human macrophages and HSCs, as well as both male and female animals, is therefore required to establish the generalizability and translational relevance of these findings. In addition, we did not assess whether the extent of LGALS3 upregulation varies across different etiologies of LC, such as alcohol-related liver disease, viral hepatitis, and NAFLD/NASH. Thus, whether LGALS3 expression and its macrophage/HSC-related functions are shared across different cirrhosis etiologies or show etiology-specific differences remains to be investigated in larger, well-annotated cohorts.

## 5 Conclusions

Our study revealed that LGALS3 marks a pro-inflammatory, low efferocytic capacity hepatic macrophage state and may contribute to LC progression by promoting HSC activation through inflammatory macrophage-derived signals potentially involving the candidate receptor ANXA2.

## Data Availability

The datasets presented in this study can be found in online repositories. The names of the repository/repositories and accession number(s) can be found in the article/**Supplementary Material**.
