# Supplementary material for: LGALS3 marks a pro-inflammatory, efferocytosis-low hepatic macrophage state and promotes hepatic stellate cell activation through the candidate receptor ANXA2 in liver cirrhosis
Source: Front Immunol. 2026 Sep 21;17:1891906. doi: 10.3389/fimmu.2026.1891906 (PMC13634827; doi:10.3389/fimmu.2026.1891906)
Supplement: Supplementary file 1 [file SupplementaryFile1.docx]

LGALS3 marks a pro-inflammatory, efferocytosis-low hepatic macrophage state and promotes hepatic stellate cell activation through the candidate receptor ANXA2 in liver cirrhosis

**Authors:** Fengchun Li¹†, Hongliang Chen¹†, Jihan Qi^1^, Jiaxin Wang^1^, Lingyi Xu^1^, Zhuo Yu^1^, Ram Prasad Chaulagain^1^, Cuihua Li^2^, Xin Ni^2^, and Shizhu Jin^1*^

†These authors contributed equally to this work and share first authorship

**Supplementary information**

**Fig. S1** Schematic illustration of mouse BDL model establishment.

**Fig. S2** (A) The location of key genes on chromosomes. (B) Correlation heatmap of Key Genes. (C) The difference in ESTIMATE score, immune score and stromal score between the normal and cirrhotic groups. (D) Heatmap of differential analysis among cell populations. (E) Violin plot of LGALS3 expression across cell populations in the normal and cirrhotic groups. (F) The circle diagrams of the differential number of interactions and interaction strength in the normal and cirrhotic groups. (G) The heatmaps of the differential number of interactions and interaction strength in the normal and cirrhotic groups. **P* < 0.05; ***P* < 0.01; ****P* < 0.001.

**Fig. S3** (A–E) Donor-level aggregation analysis. (A) Donor-level comparison of the mean LGALS3 expression in MPs between the normal and cirrhotic groups (n = 5 donors per group). (B) Donor-level comparison of the mean LGALS3 expression in inflammatory macrophages between the normal and cirrhotic groups (n = 5 donors per group). (C) Donor-level comparison of the mean AUCell scores of MPs between the normal and cirrhotic groups (n = 5 donors per group). AUCell and Scissor analyses was performed using the efferocytosis-related gene set after excluding LGALS3. **(D)** Comparison of the mean efferocytic capacity between inflammatory and non-inflammatory macrophages in cirrhotic donors. **(E)** Comparison of the mean extracellular matrix (ECM) activity between LGALS3-low and LGALS3-high cells in cirrhotic donors. (F-J) Cell-level LGALS3-excluded sensitivity analysis. (F) UMAP visualization of the AUCell score distribution for the efferocytosis-related gene set. (G) Comparison of efferocytic capacity in MPs between the normal and cirrhotic groups. (H) Comparison of efferocytic capacity between inflammatory and non-inflammatory macrophages in the cirrhotic group. (I) UMAP plot of the scissor analysis in the cirrhotic group. (J) Cell proportion diagram of the scissor analysis in inflammatory and non-inflammatory macrophages in the cirrhotic group. Of note, the comparison in panel C was performed at the donor level, whereas the corresponding comparison in panel G was performed at the cell level. **P* < 0.05; ***P* < 0.01; ****P* < 0.001.

**Fig. S4** (A) Boxplot of ANXA2 expression in samples with different fibrosis stages. (B) Boxplot of PTPRK expression in samples with different fibrosis stages. (C) Boxplot of MCAM expression in samples with different fibrosis stages. (D) Boxplot of ENG expression in samples with different fibrosis stages. (E) Comparison of ANXA2 expression in HSCs between the normal and cirrhotic groups. (F) Comparison of MCAM expression in HSCs between the normal and cirrhotic groups.

**Table S1.** The information of all the datasets in the study.

**Table S2.** Efferocytosis related genes (EFRGs).

**Table S3.** ECM related genes.

**Table S4.** Cell markers of each cell population.

**Table S5.** The available clinical characteristics of the six donors.

**Table S6.** siRNA sequences used for LGALS3 knockdown.

**Table S7.** GO enrichment analysis of upregulated genes.

**Table S8.** GO enrichment analysis of downregulated genes.

**Table S9.** KEGG enrichment analysis of upregulated genes.

**Table S10.** KEGG enrichment analysis of downregulated genes.

**Table S11.** The locations of candidate genes on human chromosomes.

**Table S12.** Bootstrap validation results of LDA.

**Table S13.** LGALS3 differential expression across cell types.

**Table S14.** Comparison of the total number of TUNEL^+^ cells between the sham and BDL groups.

**Table S15.** AlphaFold complex structure prediction between LGALS3 and predicted receptors.
